# Physical performance and maximum tongue pressure associated with oral intake independence: a retrospective study on hospitalized patients with heart failure

**DOI:** 10.1038/s41598-022-21968-w

**Published:** 2022-11-03

**Authors:** Junichi Yokota, Ren Takahashi, Ryunosuke Endo, Takaaki Chiba, Kosuke Sasaki, Keisuke Matsushima

**Affiliations:** 1 Division of Comprehensive Rehabilitation Sciences, Hirosaki University Graduate School of Health Sciences, 66-1, Hon-cho, Hirosaki, Aomori, 036-8564 Japan; 2 Department of Clinical Research, National Hospital Organization Sendai Medical Center, Sendai, Japan; 3 Department of Rehabilitation, National Hospital Organization Sendai Medical Center, Sendai, Japan

**Keywords:** Geriatrics, Disability

## Abstract

Dysphagia in patients with heart failure leads to poorer outcomes during hospitalization and after discharge. Therefore, addressing dysphagia is critical for improving patient prognosis. This retrospective observational study aimed to evaluate associations between improvements in swallowing dysfunction at the time of hospital discharge and the physical function, cognitive function, nutritional status, and maximum tongue pressure (MTP). Overall, 111 patients who underwent cardiac rehabilitation and were deemed to have oral intake impairment were included. The exclusion criteria comprised the following: pre-admission diagnosis of dysphagia, in-hospital death, and missing data. Patients were categorized based on whether they did (n = 65) or did not (n = 46) exhibit improvements in oral intake impairment, which were determined from the functional oral intake scale (FOIS) score at discharge. Associations between potential explanatory variables and the FOIS score at discharge were assessed using a linear regression model. After adjusting for covariates, such as age, sex, heart failure severity, short physical performance battery score, Mini-Mental State Examination score, transthyretin level, and provision of swallowing therapy, the FOIS score at discharge was significantly associated with the MTP (*P* = 0.024, confidence interval: 0.006–0.046). In conclusion, the MTP was independently associated with improvements in FOIS in patients with heart failure.

## Introduction

The rapid increase in heart failure (HF) incidence worldwide has been referred to as the “HF pandemic”^1^. This issue is especially pertinent in Japan due to its aging population. It has been estimated that the number of outpatients with left ventricular dysfunction will exceed 1.3 million^2^ and the incidence of HF onset in the older population will exceed 0.35 million per year by 2030^3^.

The number of patients with various non-cardiac comorbidities is also anticipated to increase as the population ages^4^. Typical non-cardiac comorbidities in older patients with HF include anemia, diabetes mellitus, chronic kidney disease, and chronic obstructive pulmonary disease^4^. However, recent evidence has indicated that dysphagia is at least as prevalent as these comorbidities^5^. Nutritional risk and cognitive dysfunction have been established as predictors of dysphagia in patients hospitalized for HF^5^. Conversely, a wide range of other factors, including oral^6^ and physical frailty^7^, maximum tongue pressure (MTP)^8^, lower limb performance, and skeletal muscle strength^9,10^, have been associated with the swallowing function in institutionalized older adults, patients with subacute stroke, older outpatients, and even frail community-dwelling older adults.

Onset of dysphagia during acute care of patients with HF has been shown to hinder improvements in the activities of daily living (ADL)^11^ and lead to poorer outcomes throughout hospitalization^12^ and after discharge^13^. Therefore, to improve the prognosis of patients with HF, reducing the incidence of dysphagia and delivering appropriate treatment to those who develop it during hospitalization is important. Unlike diseases of the central nervous system, such as stroke, HF does not directly affect the swallowing center or somatosensory system. Given this, we hypothesized that the level of oral intake independence in patients with HF was related to dysfunction of the tongue, an important component in swallowing movements. Particularly, dysphagia in HF is presumed to be caused by increased myolysis of the tongue due to inflammation. It has been reported that patients with HF have increased levels of tissue necrosis factor-α (TNF-α)^14^, interleukin (IL)-6, and TNF-α receptors^15^ in their blood. Studies also show that inflammation induces tongue muscle atrophy^16^. Furthermore, patients with HF are often frail^17^, and MTP has been reported to be reduced in frail individuals with low physiological functional reserves^18^. Several previous studies^6,7,9,10^ have also indicated relationships between the swallowing function and the physical function, cognitive function, and nutritional status. Thus, it is necessary to evaluate not only the MTP, but also a wide range of other factors related to the swallowing function to clarify their impact on the level of oral intake independence in patients with HF. Elucidation of factors associated with an improved swallowing function may, in turn, facilitate the development of interventions aimed at enhancing the ADL, quality of life, and overall prognosis. Therefore, the aim of this study was to evaluate associations between improvements in the level of oral intake by the time of hospital discharge and the physical function, cognitive function, nutritional status, and MTP.

## Methods

### Study design and patients

**Figure 1:**
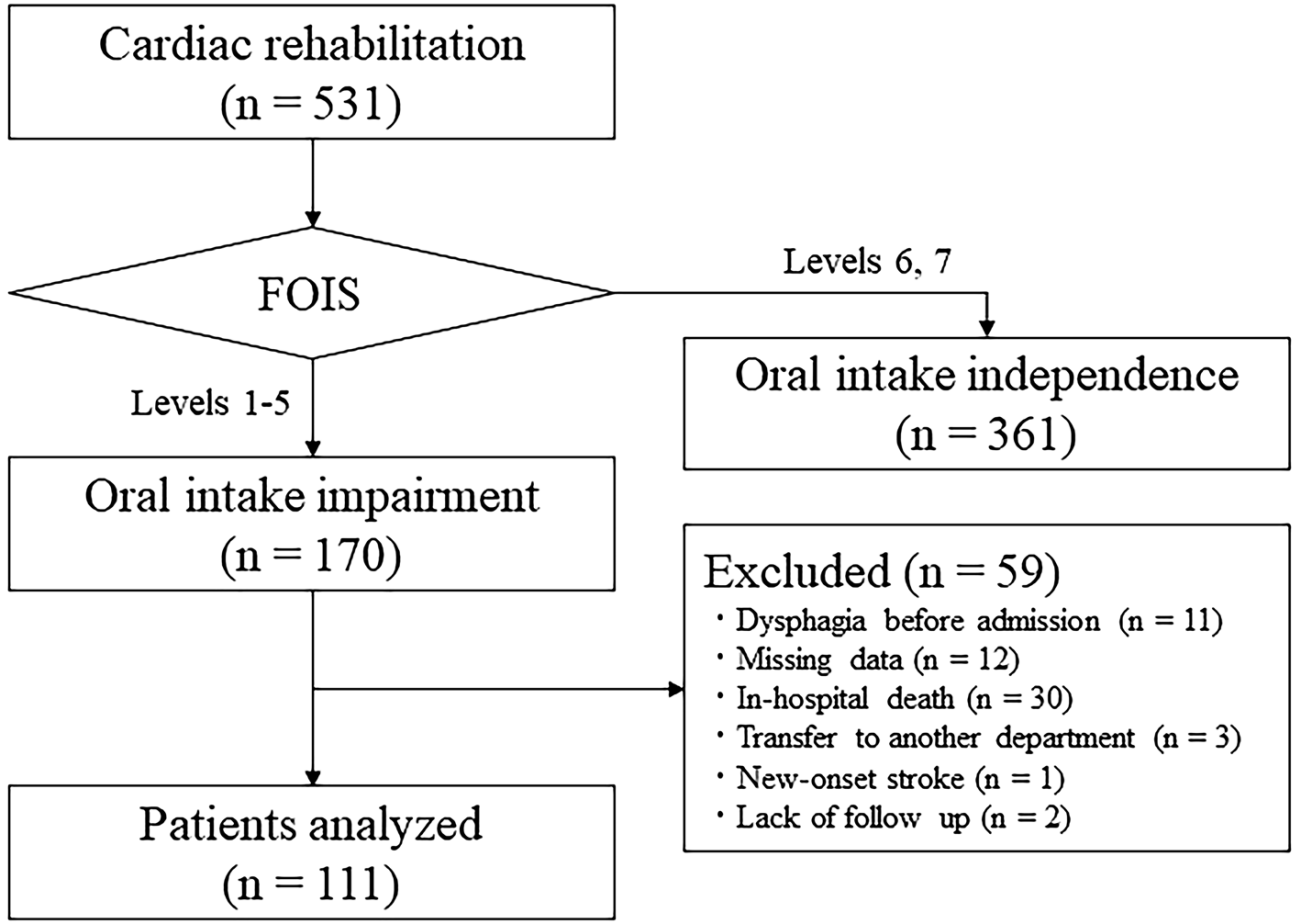
Flow diagram depicting patient selection for this study. In this study, 531 patients who received cardiac rehabilitation were classified into the oral intake impairment and non-oral intake impairment groups in accordance with their FOIS scores at rehabilitation initiation. FOIS: functional oral intake scale.

This single-center, retrospective, observational study included in-patients who were admitted to the National Hospital Organization Sendai Medical Center (an acute care hospital) between April 2016 and March 2021. Patients were included if they met the following criteria: (i) diagnosis of HF according to Japanese guidelines and admission to the institution’s Department of Cardiology, (ii) cardiac rehabilitation during hospitalization, and (iii) onset of oral intake impairment following hospital admission. Among 531 patients who underwent phase I and early phase II cardiac rehabilitation, 170 were judged to have oral intake impairment at the commencement of cardiac rehabilitation. A total of 59 patients among these were excluded due to dysphagia before admission (n = 11), missing data (n = 12), in-hospital death (n = 30), transfer to another department (n = 3), onset of stroke during hospitalization (n = 1), and lack of follow up (n = 2). Thus, 111 patients were included in the final analysis (Fig. 1).

This study was conducted in accordance with the Declaration of Helsinki and the Japanese Ethical Guidelines for Clinical Studies. The study protocol was approved by the Ethics Committee of the Sendai Medical Center (approval no.: 21-5) and the Ethics Committee of the Hirosaki University Graduate School of Medicine (approval no.: T2021-001). Since the research plan was disclosed to the public on the Sendai Medical Center’s website, obtaining informed consent was formally waived by the respective ethics committees. However, patients were allowed to refuse participation and could opt-out at any point using the institutional website; if the patient or their family expressed a clear refusal, they would be excluded from the study.

### Data extraction

Data on all variables were extracted from the electronic medical records and entered into a new database. The following data were analyzed: (i) basic characteristics (age, sex, body mass index [BMI], HF etiology, comorbidities, and medications at commencement of cardiac rehabilitation); (ii) clinical and laboratory data on admission (New York Heart Association [NYHA] functional class^19^, blood chemistry data, and ejection fraction); (iii) ADL; (iv) feeding status and swallowing function; and (v) physical function, nutritional status, cardiac rehabilitation status, and discharge disposition.

### Definitio of oral intake impairment and evaluation of the swallowing function

The level of oral intake independence was clinically evaluated using the Functional Oral Intake Scale (FOIS)^20^. The FOIS is a 7-point scale that assesses swallowing function by measuring the level of independent functional oral intake. Because it is noninvasive, the FOIS can be used for all patients, regardless of their clinical condition. In the present study, FOIS scores were determined based on the level of independent functional oral intake indicated in the electronic medical records. Specifically, this parameter was assessed by physicians, trained nurses, and speech and swallowing therapists with the use of the repetitive saliva swallowing test (RSST)^21^, the water swallowing test (WST)^22^, and/or physical assessment during food intake. The assessors observed the following aspects regarding the patient's condition during swallowing to determine the level of independent functional oral intake: extraoral loss, oral transit time, nasal reflux, oral residue, multiple swallows per bolus, laryngeal elevation, cervical auscultation, oxygen saturation, voice quality, coughing, and choking. Oral intake impairment was defined as the requirement for an oral diet modification (FOIS score ≤ 5)^11,12,23^.

The MTP was used as an index of tongue function and was measured using the JMS tongue pressure measurement device, TPM-02 (JMS, Hiroshima, Japan); this device consists of an air-filled bulb. Measurements were performed once the calibration of the inner‐balloon pressure was stabilized at 19.6 kPa. This calibration was automatically performed by the instrument, and 0.0 kPa was shown on the display screen upon successful calibration. Patients were instructed to compress the balloon (attached to the tip of the probe) between their tongue and the anterior hard palate with maximum voluntary effort. The MTP was measured thrice, and the maximum value was recorded^24^. Normal MTP was defined by the following MTP cutoff values for the Japanese population: < 30 kPa in patients aged < 75 years, < 25.8 kPa in patients aged 75–84 years, and < 19.0 kPa in patients aged > 85 years^25^. These evaluations were conducted on the first day of the rehabilitation intervention and at hospital discharge.

### Primary outcome

The primary outcome was the relationship between the FOIS score at discharge and the MTP.

### Measurement of activities of daily living, physical and cognitive function, and nutritional status

ADL was evaluated using the Barthel index (BI)^26^, which has scores ranging from 0 (full dependence) to 100 (full independence). Physical function was evaluated using a short physical performance battery (SPPB)^27^ and handgrip strength assessment^28^. The SPPB consists of balance, gait, and 5-time chair standing tests; scores range from 0 to 12 points, with higher scores indicating better physical function. The Mini-Mental State Examination (MMSE) scores^29^ and serum transthyretin levels were used as indices of cognition and nutritional risk, respectively. ADL, physical function, and cognitive function were evaluated by physiotherapists and occupational therapists on the first day of rehabilitation intervention and at hospital discharge.

### Cardiac and swallowing rehabilitation during hospitalization

Once the patient’s condition was confirmed as stable and cardiac rehabilitation was determined to be feasible by an attending physician, it was initiated under the supervision of a physiotherapist in accordance with the guidelines of the Japanese Circulation Society^30^. Cardiac rehabilitation during hospitalization consisted of two phases. Phase I consisted of mobilization (e.g., sitting on the edge of the bed or sitting in a wheelchair), standing, walking, and low-intensity resistance training. Early phase II comprised aerobic exercise and resistance training, which were adapted to individual functional deficits in each domain in accordance with standardized protocols^31^. These activities were supervised by trained physiotherapists, who used specific milestones for determining progression. During the exercise sessions, breaks were allowed as needed and a one-on-one supervision was provided by the physiotherapists in charge. As performance improved, patients advanced through a structured, gradual progression that consisted of additional small increments in exercise. Daily standardized reassessment of performance was conducted in each domain to guide exercise progression.

Swallowing rehabilitation was customized according to the patient's swallowing ability and function; it involved multidisciplinary collaboration among speech and swallowing therapists and the ward staff, oral management, indirect exercise (without food), and direct exercise (with food). Speech and swallowing therapists conducted each rehabilitation session for approximately 20–30 min. During direct exercise, small volumes of controlled foods were provided for swallowing training, and the use of auxiliary tools (straws, spoons, or glasses) was allowed. During indirect exercise, oral motor training and tongue and lip exercises were conducted for sensorimotor recovery. Alternative treatment procedures, including postural changes (e.g., head rotation and maintenance of an upright posture), modification of food consistency, changes in food volume and presentation tempo, and specific swallowing techniques (e.g., supraglottal swallowing), were also employed as necessary to ensure safe oral feeding and consequent elimination of alternative feeding methods.

### Sample size

The sample size required for linear regression model was calculated using the G*Power 3.1.9.7 software (Heinrich Heine University, Düsseldorf, Germany)^32^. As only a few previous studies have reported on the course of oral intake impairment in patients with HF, we set the effect size and the ratio of patients in whom oral intake would improve to those in whom oral intake would not improve to 0.8 and 1:1, respectively. Thus, the required sample size was determined to be 114, based on the following parameters: effect size = 0.15 [moderate]^33^, α error = 0.05, power = 0.80, and number of independent variables = 9.

### Statistical analysis

All data are reported as medians and interquartile ranges for continuous variables and as counts and percentages for categorical variables. Oral intake impairment at the start of cardiac rehabilitation was defined by a FOIS score of ≤ 5^11,12,22^. Based on whether oral intake improved, the patients were categorized into the improvement group (FOIS score = 6–7) or the non-improvement group (FOIS score = 1–5) for further analysis. In addition, a subgroup analysis was performed to investigate factors other than the MTP associated with oral intake improvement; this analysis compared patients with and without oral intake improvement among those with a normal MTP.

Continuous and categorical variables were compared between the groups using the Mann–Whitney U test and the Chi-square test, respectively. A linear regression model was used to evaluate the association between potential predictive factors and the FOIS score at hospital discharge. From a clinical perspective, the covariates selected to adjust bias were age, sex, NYHA functional class, N terminal pro-B-type natriuretic peptide (NT-proBNP) level, SPPB score, MMSE score, transthyretin level, and provision of swallowing therapy. Spearman's rank correlation coefficients were used to examine the correlation coefficient among the independent variables. Prior to the multivariate analysis, the variance inflation factor (VIF) was used to check for multicollinearity.

To clarify the associations between the presence and absence of oral intake impairment at baseline and HF severity, a bivariate analysis was performed on patients with and without oral intake impairment. In addition, multivariate logistic regression analysis was also performed with oral intake impairment at baseline as the dependent variable. The following were selected as independent variables from a clinical perspective: age, sex, BMI, NYHA functional class, NT-proBNP, hemoglobin, C-reactive protein, BI, SPPB score, MMSE score, and transthyretin level at rehabilitation initiation. All analyses were performed using JMP 14.1.0 (SAS Institute, Cary, NC, USA). A two-tailed *P* value < 0.05 indicated statistical significance.

## Results

### Baseline characteristics

The patients’ baseline demographic and other characteristics are shown in Table 1. At hospital discharge, improvement in oral intake was observed among 65 of 111 patients (59%). Patients in the improvement group had greater weights (*P* = 0.022), higher BMIs (*P* = 0.022), and higher BIs before admission (*P* < 0.001). At cardiac rehabilitation initiation and hospital discharge, the FOIS score (*P* = 0.032 and *P* < 0.001, respectively), RSST result (*P* = 0.018 and *P* < 0.001, respectively), MTP (*P* = 0.018 and *P* < 0.001, respectively), BI (*P* = 0.033 and *P* < 0.001, respectively), and physical and cognitive function were significantly better in the improvement group than in the non-improvement group. Compared to the improvement group, the non-improvement group had a significantly lower WST score (*P* < 0.001) and transthyretin level (*P* < 0.001), and had a significantly higher swallowing therapy-provision rate (*P* = 0.027) at hospital discharge. Furthermore, the proportion of patients discharged home was also lower in the non-improvement group than in the improvement group (*P* = 0.011).

**Table 1:** Baseline Characteristics.

	Crude analysis				Subgroup analysis: Patients with normal MTP			
	Over all	Improvement	Non-improvement	*P* value	Over all	Improvement	Non-improvement	*P* value
	N = 111	N = 65	N = 46		N = 61	N = 45	N = 16	
Age, years	85 (80–90)	85 (79–90)	86 (83–90)	0.415	87 (81–90)	87 (80–90)	87 (85–91)	0.410
Female, n (%)	70 (63.1)	41 (63.1)	29 (63.0)	0.997	38 (62.3)	29 (64.4)	9 (56.3)	0.561
Height, cm	154 (147–162)	155 (147–163)	152 (145–160)	0.311	152 (147–160)	154 (147–161)	151 (143–160)	0.403
Weight, kg	51 (44–61)	54 (45–65)	50 (40–56)	0.022	52 (44–60)	52 (45–63)	51 (40–58)	0.276
BMI, kg/m^2^	21.5 (19.4–24.0)	22.4 (19.5–25.7)	20.4 (17.8–22.6)	0.022	21.8 (19.5–24.5)	22.4 (19.5–25.0)	20.4 (19.5–22.0)	0.154
**Aetiology of heart failure**								
IHD, n (%)	30 (27.0)	16 (24.6)	14 (30.4)	0.496	17 (27.9)	12 (26.7)	5 (31.3)	0.725
VHD, n (%)	33 (29.7)	19 (29.2)	14 (30.4)	0.891	16 (26.2)	12 (26.7)	4 (25.0)	0.959
HHD, n (%)	16 (14.4)	10 (15.4)	6 (13.0)	0.729	11 (18.0)	9 (20.0)	2 (12.5)	0.503
Cardiomyopathy, n (%)	10 (9.0)	5 (7.7)	5 (10.9)	0.565	4 (6.6)	4 (8.9)	0 (0.0)	0.217
CHD, n (%)	1 (0.9)	0 (0.0)	1 (2.2)	0.232	0 (0.0)	0 (0.0)	0 (0.0)	1.000
Arrhythmia, n (%)	33 (29.7)	19 (29.2)	14 (30.4)	0.891	16 (26.2)	10 (22.2)	6 (37.5)	0.233
**Medical history**								
Cerebrovascular disease, n (%)	20 (18.0)	8 (12.3)	12 (26.1)	0.063	8 (13.1)	5 (11.1)	3 (18.8)	0.437
Neuromuscular disease, n (%)	2 (1.8)	1 (1.5)	1 (2.2)	0.804	1 (1.6)	0 (0.0)	1 (6.3)	0.091
Respiratory disease, n (%)	13 (11.7)	10 (15.4)	3 (6.5)	0.153	9 (14.8)	8 (17.8)	1 (6.3)	0.264
Cancer, n (%)	21 (18.9)	12 (18.5)	9 (19.6)	0.884	14 (23.0)	10 (22.2)	4 (25.0)	0.822
Diabetes, n (%)	35 (31.5)	20 (30.8)	15 (32.6)	0.837	16 (26.2)	14 (31.1)	2 (12.5)	0.146
Hypertension, n (%)	63 (56.8)	37 (56.9)	26 (56.5)	0.966	41 (67.2)	28 (62.2)	13 (81.3)	0.164
Dyslipidemia, n (%)	20 (18.0)	12 (18.5)	8 (17.4)	0.885	11 (18.0)	7 (15.6)	4 (25.0)	0.399
**Medication**								
ACEI, n (%)	17 (15.3)	11 (16.9)	6 (13.0)	0.576	12 (19.7)	9 (20.0)	3 (18.8)	0.914
ARB, n (%)	28 (25.2)	15 (23.1)	13 (28.3)	0.536	18 (29.5)	11 (24.4)	7 (43.8)	0.146
Statin, n (%)	16 (14.4)	10 (15.4)	6 (13.0)	0.729	12 (19.7)	8 (17.8)	4 (25.0)	0.533
Calcium antagonists, n (%)	35 (31.5)	21 (32.3)	14 (30.4)	0.834	17 (27.9)	12 (26.7)	5 (31.3)	0.776
Diuretics, n (%)	103 (92.8)	61 (93.9)	42 (91.3)	0.610	59 (96.7)	43 (95.6)	16 (100.0)	0.391
Digitalis, n (%)	18 (16.2)	5 (7.7)	2 (4.4)	0.475	5 (8.2)	4 (8.9)	1 (6.3)	0.704
Coronary vasodilator, n (%)	18 (16.2)	10 (15.4)	8 (17.4)	0.778	9 (14.8)	8 (17.8)	1 (6.3)	0.741
Beta-blockers, n (%)	32 (28.8)	22 (33.9)	10 (21.7)	0.165	22 (36.1)	17 (37.8)	5 (31.3)	0.641
**Clinical and laboratory findings**								
NYHA class III/IV, n (%)	99 (89.2)	57 (87.7)	42 (91.3)	0.546	52 (85.2)	38 (84.4)	14 (87.5)	0.767
Ejection fraction, %	61 (37–70)	61 (35–70)	61 (41–71)	0.587	60 (36–67)	60 (35–68)	60 (36–66)	0.776
NT-proBNP, pg/mL	5,569 (2,092–10,603)	4,820 (1,866–10,254)	6,192 (2,729–11,305)	0.269	5,569 (2,343–9,813)	5,569 (2,084–9,191)	5,651 (2,504–13,399)	0.000
Hemoglobin, g/dL	11 (10–13)	11 (10–13)	11 (10–12)	0.467	11 (10–12)	11 (10–12)	10 (9–12)	0.241
eGFR, mL/min/1.73m^2^	40 (27–59)	41 (24–60)	40 (30–59)	0.820	43 (29–58)	41 (25–58)	44 (34–56)	0.539
CRP, mg/dL	0.9 (0.2–3.5)	0.7 (0.2–2.1)	1.7 (0.3–4.2)	0.072	0.7 (0.2–2.8)	0.7 (0.3–3.6)	0.6 (0.2–2.0)	0.435
**ADL**								
BI before admission, score	85 (60–100)	90 (73–100)	68 (40–86)	< 0.001	90 (70–100)	90 (80–100)	80 (66–100)	0.089
BI at rehabilitation start, score	15 (5–40)	20 (5–45)	10 (5–30)	0.033	25 (5–48)	35 (5–53)	25 (5–34)	0.288
BI at discharge, score	60 (30–85)	75 (60–90)	35 (15–56)	< 0.001	70 (53–90)	75 (63–93)	40 (21–75)	0.001
**Swallowing status**								
** Feeding situation**								
FOIS before admission, level	7 (7–7)	7 (7–7)	7 (6–7)	0.076	7 (7–7)	7 (7–7)	7 (6–7)	0.368
Denture fitting, n (%)	62 (55.9)	39 (60.0)	23 (50.0)	0.296	41 (67.2)	30 (66.7)	11 (68.8)	0.879
Drinking start, day	2 (1–3)	1 (1–3)	2 (1–4)	0.218	1 (1–3)	1 (1–3)	2 (1–4)	0.560
Eating start, day	3 (1–5)	3 (1–5)	3 (1–6)	0.918	3 (1–5)	3 (1–6)	2 (1–4)	0.524
**At rehabilitation start**								
FOIS, level	3 (3–4)	4 (3–5)	3 (2–4)	0.032	3 (3–5)	4 (3–4)	3 (2–5)	0.742
MTP, kPa	18.7 (11.7–27.4)	21.3 (11.9–29.5)	16.3 (10.7–22.0)	0.018	24.8 (17.9–31.2)	26.1 (17.9–31.5)	23.9 (15.3–29.0)	0.403
RSST positive, n (%)	67 (60.4)	32 (49.2)	35 (76.1)	0.004	32 (52.5)	22 (48.9)	10 (62.5)	0.349
WST, score				0.058				0.373
1	1 (0.9)	0 (0.0)	1 (2.2)		0 (0.0)	0 (0.0)	0 (0.0)	
2	2 (1.8)	1 (1.5)	1 (2.2)		0 (0.0)	0 (0.0)	0 (0.0)	
3	63 (56.8)	34 (52.3)	29 (63.0)		35 (57.4)	25 (55.6)	10 (62.5)	
4	22 (19.8)	11 (16.9)	11 (23.9)		11 (18.0)	7 (15.6)	4 (25.0)	
5	23 (20.7)	19 (29.2)	4 (8.7)		15 (24.6)	13 (28.9)	2 (12.5)	
** At discharge**								
FOIS, level	6 (5–7)	6 (6–7)	5 (4–5)	< 0.001	6 (5–7)	7 (6–7)	5 (4–5)	< 0.001
Oral intake disorder recover, day	–	11 (6–19)	–	–	–	9 (6–16)	–	–
MTP, kPa	23.3 (13.7–31.4)	28.8 (21.9–33.7)	17.5 (11.6–23.5)	< 0.001	30.7 (24.4–34.4)	32.2 (25.9–36.5)	26.5 (21.6–30.1)	0.009
RSST positive, n (%)	57 (51.4)	23 (35.4)	34 (73.9)	< 0.001	24 (39.3)	14 (31.1)	10 (62.5)	0.027
WST, score				< 0.001				0.008
1	0 (0.0)	0 (0.0)	0 (0.0)		0 (0.0)	0 (0.0)	0 (0.0)	
2	1 (0.9)	0 (0.0)	1 (2.2)		0 (0.0)	0 (0.0)	0 (0.0)	
3	37 (33.3)	13 (20.0)	24 (52.2)		16 (26.2)	9 (20.0)	7 (43.8)	
4	32 (28.8)	16 (24.6)	16 (34.8)		17 (27.9)	10 (22.2)	7 (43.8)	
5	41 (36.9)	36 (55.4)	5 (10.9)		28 (45.9)	26 (57.8)	2 (12.5)	
**Physical function, nutritional status and rehabilitation situation**								
** At rehabilitation start**								
SPPB, score	0 (1–2)	1 (1–4)	1 (0–1)	< 0.001	2 (1–5)	2 (1–5)	1 (0–1)	0.001
Handgrip strength, kg	13 (9–17)	15 (10–18)	11 (7–15)	0.005	15 (11–19)	16 (11–19)	13 (9–18)	0.151
MMSE, score	18 (13–23)	20 (15–24)	17 (12–21)	0.016	21 (16–24)	21 (15–26)	21 (16–23)	0.749
Transthyretin, mg/dL	14 (11–18)	15 (12–20)	14 (9–16)	0.069	14 (11–18)	15 (11–18)	14 (10–19)	0.706
** At discharge**								
SPPB, score	3 (1–6)	5 (2–8)	1 (0–3)	0.001	4 (2–7)	5 (3–9)	1 (0–4)	< 0.001
Handgrip strength, kg	14 (9–19)	15 (12–21)	11 (7–17)	< 0.001	15 (11–20)	15 (12–21)	12 (6–19)	0.038
MMSE, score	19 (14–25)	22 (16–27)	17 (12–22)	0.001	22 (17–27)	23 (16–28)	19 (17–26)	0.426
Transthyretin, mg/dL	16 (13–19)	18 (14–21)	15 (11–17)	< 0.001	17 (14–21)	18 (14–21)	16 (10–17)	0.009
** Implementation of rehabilitation**								
Hospital stay, day	33 (23–45)	31 (22–39)	39 (24–48)	0.086	29 (21–47)	29 (19–43)	28 (22–66)	0.634
Rehabilitation start, day	3 (2–6)	3 (2–6)	4 (2–6)	0.530	4 (2–7)	3 (2–7)	4 (3–7)	0.326
Rehabilitation session, times	28 (18–40)	26 (16–35)	32 (20–44)	0.133	24 (16–41)	24 (16–40)	23 (17–55)	0.646
Total rehabilitation time, minute	800 (500–1460)	880 (540–1490)	770 (450–1390)	0.387	800 (510–1490)	880 (540–1490)	640 (430–1735)	0.528
Provision of ST, n (%)	31 (27.9)	13 (20.0)	18 (39.1)	0.027	10 (19.4)	7 (15.6)	3 (18.8)	0.767
**Discharge disposition, n (%)**								
Home residence before admission	99 (89.2)	61 (93.9)	38 (82.6)	0.060	55 (90.2)	42 (93.3)	13 (81.3)	0.163
Home	55 (49.5)	40 (61.5)	15 (32.6)	0.011	35 (57.4)	30 (66.7)	5 (31.3)	0.048
Rehabilitation hospital	31 (27.9)	14 (21.5)	17 (37.0)		14 (23.0)	8 (17.8)	6 (37.5)	
Nursing care facilities	25 (22.5)	11 (16.9)	14 (30.4)		12 (19.7)	7 (15.6)	5 (31.3)	

Values are median (interquartile range) or numbers of subjects per group (n) with percentages.

*ADL* Activities of daily living , *ACEI* Angiotensin converting enzyme inhibitor, *ARB* Angiotensin receptor blocker, *BI* Barthel index, *BMI* Body mass index, *CHD* Congenital heart disease, *CRP* C-reactive protein, *eGFR* Estimated glomerular filtration rate, *FOIS* Functional oral intake scale, *HHD* Hypertensive heart disease, *IHD* Ischemic heart disease, *MMSE* Mini-mental state examination, *MTP* Maximum tongue pressure, *NT-proBNP* N-terminal pro-B-type natriuretic peptide, *NYHA* New York heart association, *RSST* Repetitive saliva swallowing test, *SPPB* Short physical performance battery, *ST* Swallowing therapy, *VHD* Valvular heart disease, *WST* Water swallowing test.

Data are presented as median (interquartile range [IQR]), or percentage for variables.

Normal MTP was defined as the MTP value less than 30 kPa in the aged < 75, 25.8 kPa in the aged 75–84, and 19.0 kPa in the aged > 85 years.

### Factors associated with functional oral intake scale scores at discharge

No significant multicollinearity was observed among the following nine independent variables evaluated for their association with the FOIS scores at hospital discharge (Tables 2 and 3): basic characteristics (age, sex, NYHA functional class, and NT-proBNP level), variables at hospital discharge (the MTP, SPPB score, MMSE score, and transthyretin level), and provision of swallowing therapy. All VIF values were below 10, and the mean VIF was 1.273; this indicated that there was no collinearity in the model. The factors associated with the FOIS score at discharge are shown in Table 3. In the linear regression model, the FOIS score at discharge was significantly associated with MTP (*P* = 0.024, confidence interval: 0.003–0.046) even after adjusting for covariates, such as age, sex, NYHA functional class, NT-proBNP level, SPPB score, MMSE score, transthyretin level, and provision of swallowing therapy.

**Table 2:** Correlation matrix of Spearman rank-order correlation among variables in crude model.

	Variables	1	2	3	4	5	6	7	8	9
1	MTP	–	− 0.075 (0.436)	0.058 (0.548)	0.072 (0.453)	− 0.067 (0.484)	0.467 (< 0.001)†	0.408 (< 0.001)†	0.262 (0.005)*	0.180 (0.059)
2	Age		–	− 0.132 (0.168)	− 0.198 (0.038)*	0.005 (0.963)	− 0.192 (0.044)*	− 0.245 (0.010)*	− 0.325 (0.001)*	0.142 (0.137)
3	Sex			–	0.034 (0.722)	− 0.002 (0.983)	0.091 (0.342)	0.031 (0.747)	0.012 (0.010)	− 0.189 (0.047)
4	NYHA (III/IV)				–	0.022 (0.821)	0.040 (0.675)	0.091 (0.344)	0.052 (0.059)	0.152 (0.111)
5	NT-proBNP					–	0.020 (0.838)	− 0.078 (0.416)	− 0.138 (0.137)	− 0.142 (0.138)
6	SPPB						–	0.386 (< 0.001)†	0.302 (0.047)*	0.195 (0.040)*
7	MMSE							–	0.243 (0.111)*	0.119 (0.214)
8	Transthyretin								–	0.040 (0.675)
9	Provision of ST									–

Values are correlation coefficient and (*P* value).

*MMSE* Mini-mental state examination, *MTP* Maximum tongue pressure, *NT-proBNP* N-terminal pro-B-type natriuretic peptide, *NYHA* New York heart association, *SPPB* Short physical performance battery, *ST* Swallowing therapy.

*P < 0.05; †*P* < 0.001.

**Table 3:** Related factors for FOIS at discharge.

	B	SE	*P*-value	95%CI (lower, upper)	VIF
MTP	0.024	0.011	0.028	(0.003, 0.046)	1.417
Age	0.029	0.012	0.024	(0.004, 0.053)	1.465
Sex (female)	0.043	0.104	0.678	(− 0.163, 0.249)	1.069
NYHA (III/IV)	− 0.005	0.165	0.977	(− 0.331, 0.322)	1.116
NT-proBNP	0.000	0.000	0.974	(0.000, 0.000)	1.064
SPPB	0.094	0.033	0.005	(0.029, 0.158)	1.503
MMSE	0.043	0.017	0.013	(0.009, 0.076)	1.367
Transthyretin	0.053	0.019	0.007	(0.015, 0.091)	1.270
Provision of ST (yes)	− 0.136	0.118	0.248	(− 0.370, 0.097)	1.186

*CI* Confidence interval, *FOIS* Functional oral intake scale, *MMSE* Mini-mental state examination, *MTP* Maximum tongue pressure, *NT-proBNP* N-terminal pro-B-type natriuretic peptide, *NYHA* New York heart association, *SE* Standard error, *SPPB* Short physical performance battery, *ST* Swallowing therapy, *VIF* Variance inflation factor.

### Subgroup analysis

Subgroup analysis indicated that there were no significant differences in the following parameters at cardiac rehabilitation initiation between the improvement and non-improvement groups: BI (*P* = 0.288), FOIS score (*P* = 0.742), MTP (*P* = 0.403), handgrip strength (*P* = 0.151), MMSE score (*P* = 0.749), and transthyretin level (*P* = 0.706). However, the SPPB score at cardiac rehabilitation initiation was significantly lower in the non-improvement group than in the improvement group (*P* = 0.001). At hospital discharge, the BI (*P* = 0.001), FOIS score (*P* < 0.001), RSST results (*P* = 0.027), WST score (*P* = 0.008), MTP (*P* = 0.009), SPPB score (*P* < 0.001), handgrip strength (*P* = 0.038), transthyretin level (*P* = 0.009), and proportion of discharged patients (*P* = 0.048) were significantly better in the improvement group than in the non-improvement group. Moreover, according to Spearman's rank correlation coefficients (Table 4), there were no significant correlations between the MTP and the SPPB score (*P* = 0.108, r = 0.208), MMSE score (*P* = 0.464, r = 0.096), and transthyretin level (*P* = 0.107, r = 0.209).

**Table 4:** Correlation matrix among variables in subgroup analysis.

	Variables	1	2	3	4
1	MTP	–	0.208 (0.108)	0.096 (0.464)	0.209 (0.107)
2	SPPB		–	0.228 (0.077)	0.249 (0.053)
3	MMSE			–	0.182 (0.162)
4	Transthyretin				–

Values are correlation coefficient and (*P* value).

*MMSE* Mini-mental state examination, *MTP* Maximum tongue pressure, *SPPB* Short physical performance battery.

### Association between the presence and absence of oral intake impairment and heart failure severity (supplementary analysis)

This analysis was performed on 491 patients with complete baseline data, comprising 155 and 336 patients’ with the presence and absence of oral intake impairment, respectively. Bivariate analysis revealed that age, proportion of female patients, ejection fraction, C-reactive protein levels, positive RSST result, and days until drinking and eating resumption were significantly higher in the presence group than in the absence group. Furthermore, height; weight; BMI; medical history of diabetes, hypertension, and dyslipidemia; usage of angiotensin receptor blockers, statins, calcium antagonists, and beta-blockers; BI at preadmission and rehabilitation initiation; FOIS score at preadmission and rehabilitation initiation; MTP; WST score; SPPB score; handgrip strength; MMSE score; and transthyretin level were significantly lower in the presence group than in the absence group (Supplementary Table S1). In multivariate analysis, VIF was used to check for multicollinearity. All VIF values were below 10, and the mean VIF was 1.447, indicating that there was no collinearity in the model. In addition, the analysis revealed that BI, MTP, SPPB score, and MMSE score were significant independent variables for oral intake impairment at baseline (Supplementary Table S2).

## Discussion

The present study was the first to examine factors associated with the improvement in the level of oral intake independence in patients hospitalized for HF. The results showed that the MTP was independently associated with the level of oral intake independence. Sub-analyses also suggested that the SPPB score and transthyretin level were independently associated with improvement in oral intake in patients with normal MTP.

The swallowing process is classified into four phases^34^: pre-oral, oral, pharyngeal, and esophageal. During swallowing, the tongue plays a key role in bolus formation and its transport from the oral cavity to the pharynx^35^. Thus, abnormal tongue function can cause impairment of the oral and pharyngeal phase^36^ because tongue movements are required to stimulate oropharyngeal receptors and trigger subsequent swallowing events^37^. As HF does not directly affect the swallowing center, we believe that oral intake impairment in these patients may be attributed to a decreased MTP. The low MTP observed among patients with HF in the present study may have been due to physical frailty^18^, systemic inflammation^16^, and a low nutritional status^38^. Indeed, both the SPPB score (a measure of physical frailty) and the transthyretin level (a measure of nutritional risk and inflammation) were found to be significantly associated with the FOIS score at discharge; furthermore, a significant correlation was found between both of these factors and the MTP (Table 2). The MTPs at cardiac rehabilitation initiation and discharge were 16.3 and 17.5 kPa in the non-improvement group, respectively; these were below the 20.0 kPa cut-off value used for sarcopenic dysphagia diagnosis in a previous study^39^. Therefore, oral intake impairment in hospitalized patients with HF may be attributed to a decreased MTP, which is secondary to physical frailty, low nutritional status, muscle catabolism due to systemic inflammation, and/or sarcopenia. Notably, improved oral intake independence in patients with HF requires comprehensive interventions for tongue function, physical frailty, sarcopenia, and low nutritional status.

Subgroup analysis revealed that the FOIS score, MTP, SPPB score, BI, handgrip strength, and transthyretin level at discharge were significantly lower in the non-improvement group than in the improvement group. However, no significant correlations were noted among the MTP, SPPB, and MMSE scores, and transthyretin level (Table 4); this was in contrast to the results of the general analysis of all patients, which revealed significant correlations among these parameters (Table 2). This suggested that the SPPB score, MMSE score, and transthyretin level may be directly associated with the level of oral intake independence, independent of the MTP, in patients with a normal MTP. Indeed, physical and cognitive function and nutritional status have previously been reported to be associated with the swallowing function in community-dwelling older adults^7–9^. Cognitive decline (especially cerebrovascular cognitive dysfunction) has often been associated with pharyngeal stage disorders, such as delayed swallowing reflex^40^. Malnutrition can result in swallowing dysfunction due to neuromuscular dysfunction^41^, which is caused by impairment of type I muscle fibers (a major muscle type in swallowing-related muscle groups)^42,43^. Furthermore, physical frailty is an independent predictor of dysphagia^7^.

The results of this study suggest that the MTP, SPPB score, and nutritional risk may be independently associated with the level of oral intake independence in patients with HF. Because tongue function, lower limb performance, and nutritional status are components of oral frailty, physical frailty, and sarcopenia, respectively, exercise training and nutritional therapies may be effective in treating dysphagia in patients with HF. In fact, Yoshimura et al. demonstrated that a whole-body exercise intervention in patients with post-stroke dysphagia was effective in improving sarcopenic dysphagia^10^. Therefore, cardiac rehabilitation comprising whole-body endurance exercises and resistance training may theoretically be effective in not only improving the physical function, but also in preventing and treating dysphagia in patients with HF. Furthermore, isometric lingual training and expiratory muscle strength training may improve FOIS. Robbins et al.^44^ and Rogus-Pulia et al.^45^ reported that isometric lingual training significantly improved MTP, swallowing pressure, lingual volume, and dysphagia-specific quality of life. Similarly, Pauloski et al.^46^ reported that expiratory muscle strength training significantly increased geniohyoid muscle mass and strength. Practitioners must therefore consider these MTP-specific swallowing exercise programs to improve physiological functional reserve. In this study, supplementary analyses (Supplementary Tables S1 and S2) suggested that baseline oral intake impairment may be caused by the added physical stress of an acute exacerbation of HF in patients with a low physiological functional reserve, rather than by the HF itself. As such, improvements in general physical function (including frailty) and in the strength of swallowing-related muscle groups may contribute to addressing dysphagia in patients with HF.

This study had several limitations. First, the study was single-centered, retrospective, and observational in design. As such, the generalizability of the findings and the causal relationships between MTP and the level of oral intake independence are unclear. Future multicenter prospective and interventional studies are required to address this. Second, due to the retrospective study design, ward staff-supervised swallowing rehabilitation was provided to all patients; however, speech language therapist-supervised swallowing rehabilitation was not offered to all patients. Thus, it was not possible to determine whether swallowing rehabilitation by a speech language therapist during hospitalization improved the functional outcomes. However, cardiac rehabilitation was performed for all patients and approximately 60% of these patients exhibited improvement in oral intake impairment at discharge. Therefore, addition of speech language therapist-supervised swallowing rehabilitation to a conventional cardiac rehabilitation program may result in even greater improvements in the level of oral intake independence in patients with HF. Third, the present study did not perform gold standard diagnostic tests for dysphagia (for example, videofluoroscopic and videoendoscopic swallowing examinations). It is essential that instrumented swallowing assessment is performed. However, due to the nature of the retrospective observational study design of the present study, it was not possible to examine the results of instrumented swallowing assessment. The results of this study should therefore be interpreted in terms of “oral intake impairment” and not instrument-diagnosed dysphagia. This significantly limits the findings from the present study. Thus, further clinical trials to objectively characterize patients with signs and symptoms of dysphagia are important in the future. Fourth, since this study involved inpatient observation, the extent to which the level of oral intake improvement will have clinical significance remains unclear. Long-term observation may be required to demonstrate such improvements in the prognosis of patients with HF and the level of oral intake independence.

## Conclusion

The MTP, SPPB score, and transthyretin level were independently associated with improvement in oral intake impairment during acute care in patients with HF.

## Supplementary Information

Supplementary Information.

## Data Availability

The datasets generated and/or analyzed during the current study are not publicly available because the data sets contain sensitive identifying information. Any inquiries regarding data availability for this study should be directed to the corresponding author.
