# Supplementary material for: Physical performance and maximum tongue pressure associated with oral intake independence: a retrospective study on hospitalized patients with heart failure
Source: Sci Rep. 2022 Nov 3;12:18549. doi: 10.1038/s41598-022-21968-w (PMC9633604; doi:10.1038/s41598-022-21968-w)
Supplement: Supplementary file 1 — Supplementary Information. [file 41598_2022_21968_MOESM1_ESM.docx]

| Supplementally Table S1. Comparison of baseline characteristics between the presence and absence of oral intake disorder. | | | | |
| --- | --- | --- | --- | --- |
|  | Crude analysis | | | |
|  | Over all | Presence | Absence |  |
|  | N = 491 | N = 155 | N = 336 | *P*-value |
| Age, *years* | 82 (75-87) | 85 (81-90) | 80 (73-86) | <0.001 |
| Female, *n (%)* | 268 (54.6) | 96 (61.9) | 172 (51.2) | 0.026 |
| Height, *cm* | 155 (147-163) | 153 (147-160) | 155 (148-164) | 0.028 |
| Weight, *kg* | 54 (46-64) | 50 (42-57) | 57 (48-65) | <0.001 |
| BMI, *kg/m^2^* | 22.6 (20.0-25.5) | 21.0 (18.9-23.7) | 23.5 (20.7-25.9) | <0.001 |
| Aetiology of heart failure |  |  |  |  |
| IHD, *n (%)* | 124 (25.3) | 37 (23.9) | 87 (25.9) | 0.632 |
| VHD, *n (%)* | 115 (23.4) | 36 (23.2) | 79 (23.5) | 0.945 |
| HHD, *n (%)* | 70 (14.3) | 16 (10.3) | 54 (16.1) | 0.090 |
| Cardiomyopathy, *n (%)* | 40 (8.2) | 10 (6.5) | 30 (8.9) | 0.351 |
| CHD, *n (%)* | 3 (0.6) | 1 (0.7) | 2 (0.6) | 0.943 |
| Arrhythmia, *n (%)* | 133 (27.1) | 35 (22.6) | 98 (29.2) | 0.127 |
| Medical history |  |  |  |  |
| Cerebrovascular disease, *n (%)* | 100 (20.4) | 39 (25.2) | 61 (18.2) | 0.073 |
| Neuromuscular disease, *n (%)* | 11 (2.2) | 2 (1.3) | 9 (2.7) | 0.334 |
| Respiratory disease, *n (%)* | 53 (10.8) | 16 (10.3) | 37 (11.0) | 0.819 |
| Cancer, *n (%)* | 89 (17.5) | 29 (18.7) | 57 (17.0) | 0.636 |
| Diabetes, *n (%)* | 198 (40.3) | 52 (33.6) | 146 (43.5) | 0.038 |
| Hypertension, *n (%)* | 335 (68.2) | 91 (58.7) | 244 (72.6) | 0.002 |
| Dyslipidemia, *n (%)* | 129 (26.3) | 29 (18.7) | 100 (29.8) | 0.010 |
| Medication |  |  |  |  |
| ACEI, *n (%)* | 73 (14.9) | 19 (12.3) | 54 (16.1) | 0.270 |
| ARB, *n (%)* | 178 (36.3) | 33 (21.3) | 145 (43.2) | <0.001 |
| Statin, *n (%)* | 131 (26.7) | 24 (15.5) | 107 (31.9) | <0.001 |
| Calcium antagonists, *n (%)* | 171 (34.8) | 44 (28.4) | 127 (37.8) | 0.042 |
| Diuretics, *n (%)* | 452 (92.1) | 144 (92.9) | 308 (91.7) | 0.638 |
| Digitalis, *n (%)* | 45 (9.2) | 10 (6.5) | 35 (10.4) | 0.157 |
| Coronary vasodilator, *n (%)* | 104 (21.2) | 28 (18.1) | 76 (22.6) | 0.251 |
| Beta-blockers, *n (%)* | 177 (36.1) | 45 (29.0) | 132 (39.3) | 0.028 |
| Clinical and laboratory findings | | | | |
| NYHA class III/IV, *n (%)* | 401 (81.7) | 134 (86.5) | 267 (79.5) | 0.063 |
| Ejection fraction, *%* | 54 (33-67) | 60 (35-70) | 49 (31-66) | 0.004 |
| NT-proBNP, *pg/mL* | 5,052 (2,403-11,844) | 6,832 (2,662-12,797) | 4,340 (2,352-11,682) | 0.060 |
| Hemoglobin, *g/dL* | 11 (10-13) | 11 (10-12) | 11 (10-13) | 0.069 |
| eGFR, *mL/min/1.73m^2^* | 42 (26-58) | 40 (25-58) | 42 (26-58) | 0.704 |
| CRP, *mg/dL* | 0.5 (0.2-2.0) | 1.1 (0.3-3.9) | 0.5 (0.2-1.4) | <0.001 |
| ADL |  |  |  |  |
| BI before admission*, score* | 100 (80-100) | 80 (50-100) | 100 (90-100) | <0.001 |
| BI at rehabilitation start, *score* | 55 (25-80) | 15 (5-40) | 65 (45-90) | <0.001 |
| Swallowing status |  |  |  |  |
| Feeding situation |  |  |  |  |
| FOIS before admission, *level* | 7 (7-7) | 7 (6-7) | 7 (7-7) | <0.001 |
| Denture fitting, *n (%)* | 266 (54.1) | 85 (54.8) | 181 (53.9) | 0.841 |
| Drinking start*, day* | 1 (1-2) | 1 (1-3) | 1 (1-2) | <0.001 |
| Eating start, *day* | 2 (1-3) | 3 (1-6) | 1 (1-2) | <0.001 |
| At rehabilitation start |  |  |  |  |
| FOIS, *level* | 7 (4-7) | 3 (2-4) | 7 (7-7) | <0.001 |
| MTP, *kPa* | 27.2 (20.0-33.7) | 18.7 (11.1-27.8) | 29.4 (23.6-35.1) | <0.001 |
| RSST positive, *n (%)* | 154 (31.4) | 101 (65.2) | 53 (15.8) | <0.001 |
| WST, *score* |  |  |  | <0.001 |
| 1 | 1 (0.2) | 1 (0.6) | 0 (0.0) |  |
| 2 | 2 (0.4) | 2 (1.3) | 0 (0.0) |  |
| 3 | 153 (31.1) | 97 (62.6) | 56 (16.7) |  |
| 4 | 81 (16.5) | 29 (18.7) | 52 (15.5) |  |
| 5 | 254 (51.7) | 26 (16.8) | 228 (67.9) |  |
| Physical function, nutritional status and rehabilitation situation | | | | |
| At rehabilitation start |  |  |  |  |
| SPPB, *score* | 4 (1-8) | 1 (0-2) | 5 (3-9) | <0.001 |
| Handgrip strength, *kg* | 17 (12-23) | 12 (9-16) | 19 (15-25) | <0.001 |
| MMSE, *score* | 23 (18-27) | 17 (12-22) | 25 (21-27) | <0.001 |
| Transthyretin, *mg/dL* | 16 (12-21) | 14 (10-17) | 17 (13-22) | <0.001 |
| Values are median (interquartile range) or numbers of subjects per group (n) with percentages.  ADL, Activities of Daily Living; ACEI, Angiotensin Converting Enzyme Inhibitor; ARB, Angiotensin Receptor Blocker; BI, Barthel Index; BMI, Body Mass Index; CHD, Congenital Heart Disease; CRP, C-Reactive Protein; eGFR, estimated Glomerular Filtration Rate; FOIS, Functional Oral Intake Scale; Hypertensive Heart Disease, HHD; IHD, Ischemic Heart Disease; MMSE, Mini-Mental State Examination; MTP, Maximum Tongue Pressure; NT-proBNP, N-Terminal pro-B-type Natriuretic Peptide; NYHA, New York Heart Association; RSST, Repetitive Saliva Swallowing Test; SPPB, Short Physical Performance Battery; ST, Swallowing Therapy; VHD, valvular heart disease; WST, Water Swallowing Test.  Data are presented as median (interquartile range [IQR]), or percentage for variables. | | | | |

| Supplementally Table S2. Multivariate logistic regression analyses for oral intake impairment at rehabilitation start. | | | | | | |
| --- | --- | --- | --- | --- | --- | --- |
| Variables | B | SE | Wald | *P*-value | Odds ratio | 95％CI  (lower, upper) |
| Age (+1 years) | 0.004 | 0.016 | 0.066 | 0.797 | 1.004 | (0.973, 1.037) |
| Sex (female) | 0.006 | 0.140 | 0.002 | 0.968 | 1.011 | (0.585, 1.748) |
| BMI (+1 kg/m^2^) | -0.043 | 0.030 | 2.163 | 0.141 | 0.957 | (0.904, 1.015) |
| NYHA IV (vs I-III) | -0.084 | 0.192 | 0.189 | 0.664 | 0.846 | (0.398, 1.799) |
| NT-proBNP (+1 pg/mL) | 0.000 | 0.000 | 0.090 | 0.764 | 1.000 | (1.000, 1.000) |
| Hemoglobin (+1 g/dL) | 0.123 | 0.066 | 3.542 | 0.060 | 1.131 | (0.995, 1.286) |
| CRP (+1 mg/dL) | 0.051 | 0.037 | 1.887 | 0.170 | 1.052 | (0.979, 1.131) |
| Barthel index (+5 points) | -0.170 | 0.031 | 30.702 | <0.001 | 0.843 | (0.794, 0.896) |
| MTP (+1 kPa) | -0.057 | 0.016 | 12.923 | 0.000 | 0.945 | (0.916, 0.974) |
| SPPB (+1 points) | -0.143 | 0.061 | 5.411 | 0.020 | 0.867 | (0.769, 0.978) |
| MMSE (+1 points) | -0.067 | 0.026 | 6.651 | 0.010 | 0.936 | (0.889, 0.984) |
| Transthyretin (+1 mg/dL) | -0.012 | 0.025 | 0.247 | 0.619 | 0.988 | (0.940, 1.037) |
| Model fitting: *P*<0.001, Lack of fit: *P* =1.000,  BMI, Body Mass Index; CRP, C-Reactive Protein; MMSE, Mini-Mental State Examination; MTP, Maximum Tongue Pressure; NT-proBNP, N-Terminal pro-B-type Natriuretic Peptide; NYHA, New York Heart Association; SPPB, Short Physical Performance Battery. | | | | | | |
|  |  |  |  |  |  |  |
